# Characterization of the firing behaviour of an illite-kaolinite clay mineral and its potential use as membrane support

**DOI:** 10.1016/j.heliyon.2019.e02281

**Published:** 2019-08-30

**Authors:** Abdelaziz Elgamouz, Najib Tijani, Ihsan Shehadi, Kamrul Hasan, Mohamad Al-Farooq Kawam

**Affiliations:** a Department of Chemistry, College of Sciences, Research Institute of Science and Engineering, University of Sharjah, P.O. Box 27272, Sharjah, United Arab Emirates; b Equipe Membranes, Matériaux et Procédés de Séparation, Faculté des Sciences, Université Moulay Ismaîl, Meknès, Morocco

**Keywords:** Analytical chemistry, Inorganic chemistry, Physical chemistry, Membrane supports, Illite, Kaolinite, Calcination, Surface area, Clay minerals

## Abstract

The commercial value of any clay depends on its physical and chemical properties, these could help in tuning the characteristics of ceramic membrane supports required at extreme filtration conditions. The characteristics of two clay minerals named SA and CH were studied at various firing temperatures. The composition in oxides of both raw materials consisted of quartz (44.40 ± 0.60 to 46.98 ± 0.57 m%), alumina (13.16 ± 0.56 to 19.64 ± 0.48 m%), iron oxide (4.85 ± 0.46 to 6.37 ± 0.70 m %), and relatively smaller amounts of alkaline-earth oxides (3.34 ± 0.43 to 5.98 ± 0.33 m% calcium oxide and 1.98 ± 0.18 to 5.87 ± 0.34 m% for magnesium oxide). XRD of the investigated samples indicated the presence of kaolinite and illite as pure clay fractions in the clay mineral. SEM showed that the clay minerals were constituted from fine poorly crystalline particles with particles’ size more than 5 μm. The specific surface areas of the clay minerals were found to vary between 94.5 ± 6.3 to 138.6 ± 4.2 m^2^/g using methylene blue stain test, indicating that, the clay minerals fall within chlorite, illite, and kaolinite categories. The porosity of the clay supports made from both clays were found to be maximal with values of 23.45% ± 0.66 and 21.61% ± 0.60 for SA and CH clay materials respectively at 700°C. These values were a direct result of capillary movements of water in the specimen pores that were opened to the outside leading to the highest number macropores and mesopores in the specimen.

## 1 Introduction

Clay refers to a sedimentary rock, which constitutes the major composition of the Earth's crust (Chiappone et al., 2004). Clays are composed of very fine particles which gets plasticized when mixed with appropriate amount of water and harden when dried or calcined. clays are mainly composed from large proportion of hydrated alumino-silicates which are arranged in tetrahedral or octahedral sheets connected through oxygen atoms which occupy the vertices, while aluminum and silicon are occupying the centers of the polyhedra. Silicon occupy the center of the silicon-oxygen tetrahedron, while Al^3+^, Mg^2+^ and Fe^2+^ serve as coordinating cations in the octahedral sheets formed from octahedrons that share edges composed of oxygen and hydroxyl anions giving rise to different types of clay minerals (Adeyemo et al., 2017). A lot of effort has been dedicated to give a specific definitions and classifications of clays, however, these are still disputed, mainly because clays are materials considered to have the highest variability in occurrence, texture, mineralogy and the most diverse applications in comparison to other materials (Guggenheim and Martin, 1995; Zakaria et al., 2009). The adsorption qualities of clays are directly related to the total cation exchange capacity (CEC) and specific surface area which may be affected by the pH, time of contact and temperature of the medium as well as the type and concentration of the adsorbate (Stockmeyer, 1991; Tahir and Rauf, 2006; Errais et al., 2012).

There are several types of clay mineral that occur naturally as pure minerals. However, common clay minerals usually exist in nature as mixtures of pure clay fractions like illite, kaolinite, and smectite in addition to various impurities as quartz, calcite, and organic remains, some of these materials may impart plasticity and hardness when specimens are dried or fired. From a geological point of view, common clay minerals are used to describe clay minerals of different origins, age and composition (Grim, 1968; Moore and Reynolds, 1989; Bergaya and Lagaly, 2006; Azejjel et al., 2010).

The commercial value of any clay mineral depends primarily on its physical properties such as plasticity, strength, shrinkage, vitrification range, refractoriness, color of the calcined surface, porosity and the adsorption capacity (Garzón et al., 2016; Kasprzhitskii et al., 2016). In Morocco, clay minerals are mainly used for making traditional and modern building materials such as bricks and tiles. Clay minerals are also used to make pottery crafts and kitchen wares. Clay minerals reserves in Morocco are sufficient to satisfy the demand of the ceramic industry mainly through three major sites; Safi, Salé and Fez-Meknes. Exploitation of clay minerals in Morocco is mainly artisanal and semi-industrial by family firms and craftsmen. The absence of preliminary studies of the raw clay material that is used to make clay items are responsible for deformation and defects in the final products. In this contest, many studies have been carried out on the topic of clay minerals characterization in Morocco to understand their origins and time of formation (Hajjaji et al., 2002). Given the pivotal role that pure clay fractions contained in a clay mineral could play, it is important to establish whether techniques used for clay characterization were sufficient or not. Anbri et al. (2008), characterized a clay mineral from the central region of Morocco by using X-ray diffraction, thermal analysis and FT-IR, the clay was used for the fabrication of plane membranes for microfiltration, apart from X-ray analysis which shows existence of illite and kaolinite no proper quantification of these fractions was made. The authors went on explaining the porosity of the plane membranes based on erroneous evidences. Karim et al. (2009), have used X-ray diffraction as a sole technique to characterize a clay from Safi region and proceeded to its use in the removal of Basic Red 46 from aqueous solution, without proper characterization of the clay fractions, wrong interpretations of the data can occur. El Ouahabi et al. (2014) suggested that, the differences between the central region and northern region's clays in Morocco are probably due to their cation exchange capacity (CEC). To study the CEC of a clay mineral, clay fractions must be isolated and thoroughly characterized. Sadik et al. (2014) have aimed to use a Cretaceous clay deposit (Moroccan Meseta) for the preparation of refractory products by compacting the material and centering it between 900 and 1000°C. However, choosing the right firing program requires a full investigation of the clay fraction in the clay mineral in addition, that different thermal phenomena which happen during the thermal analysis of the clay material must be identified and taken into consideration when designing a firing program (Elgamouz and Tijani, 2018, 2018a). This paper attempts to establish a correlation between Energy Disperse Spectroscopy (EDAX) elemental analysis, X-ray diffraction, thermal analysis (DTA and TGA), scanning electron microscopy (SEM), Fourier Transform Infrared Spectroscopy (FTIR) and methylene blue stain test as set by the French Association of Normalization (AFNOR) and the American Society for Testing and Materials (ASTM) (Chiappone et al., 2004). Additionally, the clay fractions were isolated from an abundantly available clay mineral sampled from Safi region, which is the center of the ceramic industry in the west-central Moroccan province and was fully characterized with the above-mentioned techniques while the clay fraction was semi-quantified by the methylene blue test, these results were validated using bentonite Nano-clay. This paper attempts to use multiple physicochemical characterization of two clay minerals to find optimum conditions where clay ceramic membrane supports could be made.

## 2 Materials and methods

### 2.1 Clay mineral sampling

The clay materials used in this study were collected from Safi region which is adjacent to Middle Atlas, two clay samples were collected named **SA** and **CH**. The site of sampling is situated at about 10 km from Safi center. (geographical coordinates: Latitude: 32° 16′ 60.00″ North and the Longitude: -9° 13′ 60.00″ West). The clay material was crushed to coarse material, then to fine powder followed by the sieving operation using standardized ASTM sieves in the range 250–315 μm.

### 2.2 Mass loss on ignition of clay

**Fig. 1:**
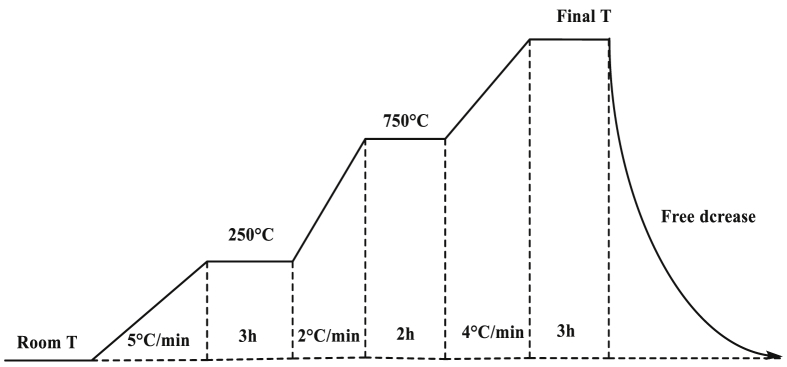
Heating program used to consolidate the clay specimens (Elgamouz and Tijani, 2018a).

Clay flat disks were prepared from clay powders with particle sizes in the range of 250–500 μm sieved using standardized sieves according to the American Standard and Testing Materials (ASTM). Preliminarily the clay material was crushed to coarse material, then to fine powder followed by the sieving operation. 3.0 g of clay powders were uniaxialy compacted in a stainless-steel mold under a pressure of $4.6\times 10^{7}$ Pa, to obtain pellets with a diameter of 25.0 mm and a thickness of 2.0 mm. The flat disks were dried in an oven at 110°C and calcined to final firing temperature ranging from 250 to 1100°C. The temperature of the oven was raised to the final temperature following the firing program described in Fig. 1. After cooling down to room temperature in a desiccator, to avoid readsorption of water, flat disks were weighed. Mass losses were determined according to Eq. (1).

(1) mass ​loss ​% ​=(m110−mTm110)×100

In Eq.(1), m_110_ is the flat disk mass at 110°C or dry clay mineral sample, m_T_ is the disk or powder masses fired to final temperature T.

### 2.3 Linear firing shrinkage

Linear firing shrinkage is a thermal method which is based on the study of expansion or shrinking of the clay mineral specimens. The compacted flat disks prepared previously were calcined to final temperature of 250, 500, 700, 800, 850, 900, 950, 1000, 1050 and 1100°C. The specimens in this study were set to a final temperature following a heating program (Fig. 1) which was used in a previous study (Elgamouz and Tijani, 2018a). Eq. (2) was used to determine shrinkage coefficients.

(2) ΔL/L0=(LT−L0L0)×100

In Eq. 2, L_0_ is the diameter of the flat disk before calcination and L_T_ is the diameter after the calcination at final temperature T.

### 2.4 Clay fraction isolation

5.0 g of ground clay was suspended in 1.0 L of deionized water and stirred vigorously for 18 h. After adding 2 drops of 10% HCl. The suspension was viscous and the pH > 7.0. HCl was drop wise added to the suspension with a speed of 2 drops/hour, a total number of 50 drops were added, the pH was found to be 7.30. At this stage, the suspension was allowed to settle for 50 min. 40.0 mL aliquot of the supernatant were placed in four tubes and centrifuged at 2700 rpm for 30 min a very small amount of solid was recovered from the bottom of each tube, they were collected in one tube then centrifuged again at 1750 rpm for 30 min the recovered solid was sent to XRD. The sample was analyzed and proved not to be the clay fraction. The rest of the supernatant in the 1.0 L beaker was discarded and the settled coarse material at the bottom of the beaker was collected in 80.0 mL deionized water then transferred to tubes and centrifuged at 3500 rpm for 40 min. The supernatants were discarded, and solids were suspended again in water, and centrifuged at 600 rpm for 7 min, this operation was carried out two times to wash the solid material. At a final step the cloudy supernatant, suspected to contain particles with size less than 2μm (clay fraction) was collected in 100 mL beaker then centrifuged at 2750 rpm for 40 min the solid collected from the tubes was sent to XRD.

### 2.5 Water absorption and porosity

Determining the porosity allows evaluating the percentile of voids in the material as well as gives an idea about the durability of the final product. Flat disks prepared in the linear shrinkage part and calcined to different final temperatures ranging from 500 to 1000°C were immersed for a period of 24 h in a beaker filled with degassed distilled water boiled for a period of 2 h. The flat disks were removed and left to dry in open air for 10 min on a filter paper, then their masses were recorded. The porosity was determined using Eq. (3).

(3) P ​% ​=(mf−m0m0)×100

In Eq.(3), m_0_ is the initial mass and m_f_ is the finale mass of the specimen.

### 2.6 Chemical resistance

Chemical resistance is a test that consists of calculating the loss of mass of a material in an acidic or basic environment. Two flat disks from both clays SA and CH were fired to 850°C were immersed in solutions adjusted to pH = 5.0 using hydrochloric acid and pH = 10.0 using sodium hydroxide and left in contact with the solution for 24 h. After the flat disks were removed from the solutions they were weighted and the resistance was calculated using Eq. (4).

(4) R ​% ​=(mo−mpHmo)×100

In Eq. (5) m_o_ is the mass of the specimen before pH attack and m_pH_, is its mass after removing the specimen from the acidic (HCl, pH = 5.0) or basic (NaOH, pH = 10.0) solutions for 24 h.

### 2.7 Methylene blue tests

**Fig. 2:**
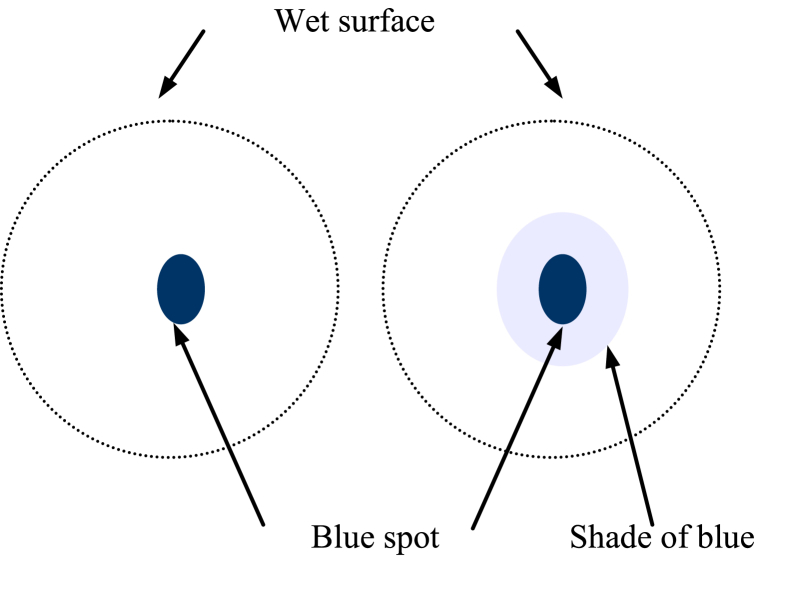
Saturation of the clay mineral by methylene blue dye using AFNOR and ASTM tests.

Methylene blue stain test according to AFNOR was used to identify the cation exchange capacity of a given soil by measuring the amount of methylene blue needed to cover the total specific surface area (internal and external) of clay minerals contained in a specific soil. To reflect the activity and have a qualitative indication of the type of the clay minerals that are present in a soil, AFNOR has defined a parameter called “blue value of soil” (V_B_). According to AFNOR (ANFOR, 1993; Chiappone et al., 2004), the recommended amount of material to be tested is based on the mineralogical composition (phyllite textures) of a soil; 30–60 g for soils rich in clay minerals and 60–120 g for those which are not. AFNOR procedure is as follows; the clay materials SA and CH were grinded in a mortar and then dried in an oven at 105°C for 12 h. Then 60.0 g of each sample was suspended in 500 mL of distilled water and stirred vigorously until it was homogenized. Flow volumes of 5.0 mL of methylene blue solution at concentration of 10.0 g/L were added to the homogenized solutions using a burette. After each addition, small quantities were sampled by a capillary tube and spotted on a filter paper. A deep blue spot surrounded with a wet colorless surface is obtained as represented in Fig. 2. The addition of methylene blue and the sampling process continued until the color of the wet surface surrounding the blue spot was turned into light blue. At this point, the saturation of the clay mineral got closer and the last addition of methylene blue was performed. Persistence of blue color was an indication of saturation which could be verified by stirring the clay mineral dispersion and collecting samples every 1 min, If the intensity of the blue spot persisted after 5 samplings, then the entire specific surface area of clay minerals presents in the soil had been saturated by the methylene blue. The clay mineral blue value (V_B_, g/100.0 g) was calculated using Eq. (5).

(5) VB=(V×0.01×100)M

In Eq. (5), V defines the methylene blue volume flowed in mL, 0.01 is the concentration in g/mL of the methylene blue solution, and M is the mass in grams of the dry sample.

In the same way, the ASTM (ASTM, 2009) defined a blue index (MIB, in equivalents/100 g) and is calculated according to Eq. (6).

(6) MIB=(E×V×0.1)W

In Eq. (6), E is the number of equivalents of methylene blue per mL, V is the volume of the methylene blue solution in mL (unit of titration is 1.0 mL), and W is the mass of the dry sample in g. The ASTM method is based on the same concept illustrated previously in the AFNOR method. However, the ASTM method addresses the mechanism of chemical adsorption and counts for the excess of negative charges. Hence, methylene blue provides the counter ions to neutralize the excess negative charge in the clay mineral.

### 2.8 X-ray diffraction analysis

X-ray diffraction (XRD) patterns were obtained with CuK_α_ radiation (*λ* = 1.540 Å) on a PW1710 Philips Analytical diffractometer controlled by XPERT Quantify software (EA Almelo, The Netherlands) operating at 40 kV and 30 mA, with a copper anode and a graphite monochromator. The Data was collected between 2*θ* = 5.0°–80° in incremental steps of 0.05° with a rate (*t)* = 0.5 s/step. A monochromator with a normal divergence (1.0°) and receiving slits of 0.1 mm dimensions were used. 1300 points were recorded using continuous scans. Non-oriented preparations of the samples were used; fine enough powders were used in order to ensure the homogeneity of the sample. This method is very reliable and gives excellent results with regards to the total recovery of the clay mineral. Powder samples consisted of a given crystalline phase always gave rise to diffraction peaks in the same directions, with the relatively constant heights. This diffraction pattern thus formed a true signature of the clayey phase. The oriented preparations of the clay fraction (<2.0 μm) were prepared through 6 steps; grinding, discharge, decarbonization, washing, sample dispersion and extraction of particles smaller than 2.0 μm as described by El Yakoubi (2006). Only the clay fraction of SA clay mineral was determined because of similarities between the two samples SA and CH.

### 2.9 Thermal analysis

The thermal differential analysis (DTA) and thermal gravimetric analysis (TGA) were performed using a thermo-balance instrument-type (SETARAM) which gave simultaneous curves of DTA and TGA. The thermal analysis measurements of **SA** clay only were performed in static air with a heating rate of 10°/min because of similarities with **CH** clay mineral.

### 2.10 FTIR analysis

FT-IR spectra were recorded using an FT-IR Bruker Platinum Spectrometer fitted with an attenuated total reflection (ATR) unit, with single reflection geometry. Intensities of the spectra were recorded in the transmittance mode. Clay mineral samples were calcined to 50, 250, 500, 700, 800, 900 and 1000°C. The clay material powders were kept at 70°C in an oven, then taken in a desiccator to FTIR (ATR) analysis. The infrared spectra of both **SA** and **CH** clay minerals were identical. Therefore, only the infrared spectra of **SA** clay mineral at different temperatures are shown. Samples were introduced to the attenuated total reflection (ATR) unit with a single reflection geometry and spectra were collected in the range 4000–400 cm^−1^ in the transmittance mode.

### 2.11 Scanning electron microscopy (SEM)

The morphology, distribution and composition of the clay material were visualized and analyzed using a Tescan VEGA XM variable pressure SEM equipped with Oxford Instruments X-Max 50 EDAX detector, controlled with AZtecEnergy analysis software with a resolution of 125 eV to determine the abundance of elements. SEM images were taken for SA only at an accelerating voltage of 30 kV controlled by VEGA TC software. The EDAX analysis were carried out in triplicates to the average value in oxide and standard deviation.

### 2.12 Statistical analysis

Organic matter content, shrinkage analysis, mass loss on ignition, porosity and methylene blue stain tests were undertaken in triplicate. The results were calculated from the averages of all sample readings and represented as Mean ± SD. Calculations were made using Excel 2016.

## 3 Results and discussion

The mass losses for both clay materials at final temperatures of 250, 500, 700, 800, 900 and 1000°C are given in Table 1. The mass loss below 500°C, was a measure that gave an idea about the quantity of products decomposed or volatilized below this temperature; such as organic matter present in the clay material as well as constitutional waters. Prior to 500°C, the mass loss was determined at 250°C as the highest loss is registered below this temperature. Values of 4.12 ± 0.19% and 3.74 ± 0.27% were found for **SA** and **CH** clay mineral samples respectively. The organic matter and constitutional waters content of both clay minerals were quit higher compared to other clay minerals from other regions in Morocco such as Tetouan, Tangier and Meknes (El Yakoubi, 2006). The increase of the firing temperature from 500°C to 700°C leads to an increase in the mass loss with a highest mass loss difference of 6.03% ± 0.13 and 6.11 ± 0.18 for both clays **SA** and **CH** respectively. Low mass loss differences were recorded for both clay materials when temperatures were increased above 700°C with a step increase of 100°C. The lowest loss difference at high temperature was recorded between 700 and 800°C with values of 0.26% ± 0.11, and 0.17 ± 0.08 for SA and CH clay materials respectively.

**Table 1:** Percent mass losses (%) of the raw clay materials at different temperatures.

	SA	CH	Loss difference (SA)	Loss difference (CH)
250°C	4.12 ± 0.19	3.74 ± 0.27	4.12 ± 0.19	3.74 ± 0.27
500°C	5.23 ± 0.09	5.05 ± 0.13	1.11 ± 0.21	1.31 ± 0.30
700°C	11.26 ± 0.09	11.17 ± 0.13	6.03 ± 0.13	6.12 ± 0.18
800°C	11.52 ± 0.06	11.33 ± 0.07	0.26 ± 0.11	0.16 ± 0.14
900°C	12.38 ± 0.05	12.28 ± 0.07	0.87 ± 0.08	0.95 ± 0.10
1000°C	12.69 ± 0.07	12.60 ± 0.07	0.31 ± 0.09	0.31 ± 0.10

**Fig. 3:**
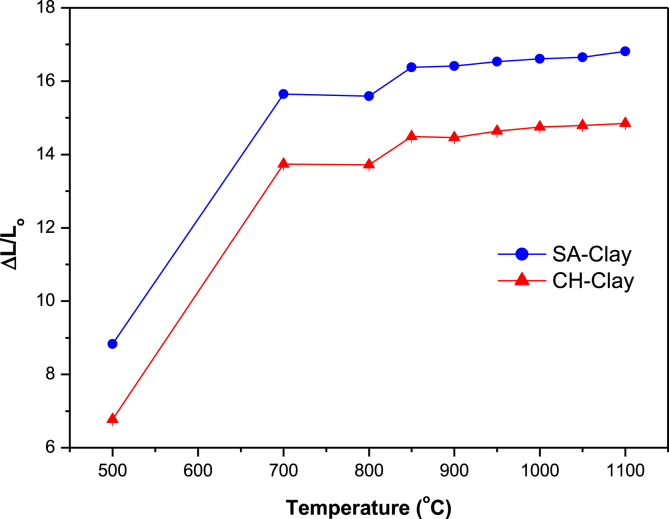
Shrinking of flat disks specimen of both clay materials SA and CH at various final calcination temperatures.

**Fig. 4:**
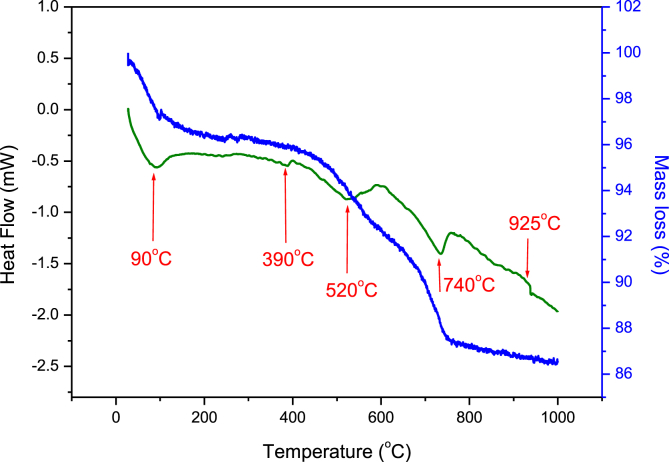
Thermal analysis of raw clay mineral (a) Thermo-gravimetric analysis and (b) differential thermal analysis (DTA) of the clay mineral.

Specimens’ shrinkage or dilatation of the two clay mineral flat disks were studied at final firing temperature of 250, 500, 700, 800, 850, 900, 950, 1000, 1050 and 1100°C. Data is represented in Fig. 3. Flat disks were found to shrink with ΔL/L_0_ values of 8.83 ± 0.07 and 6.77 ± 0.03% for SA and CH clay flat disks respectively when calcined to final temperatures of 500°C. On further increase of final firing temperatures of 700°C, a dramatic increase was observed to values of 15.65 ± 0.09 and 13.74 ± 0.04 for SA and CH clays respectively. These values remain unchanged when disks were heated to 800°C. a second most important increase was noticed when firing temperatures were increased to 850°C, at which values of 16.41 ± 0.08 and 14.46 ± 0.03 were registered for both SA and CH specimens. These remain unchanged for remaining firing temperatures. The shrinking of the clay material was explained by the fusing of the grains under the effect of heat which lead to densification of the clay material. The mass loss during calcination was also investigated at temperatures of 750, 850 and 950°C. The mass losses were resulted from the loss of structural water, oxidation of FeO, and decomposition of sulfates, carbonates and nitrates (El Gamouz et al., 2007). In general, values less than 5.0% mass loss in the mass of final products is very tolerable, and give the clay material an added value in making it cost effective material in the brick industry. The quality of the ceramic specimens which are made from the clay material was studied through water absorption. A much more understanding of the behavior of the clay materials studied was gained through thermal analysis (TGA and DTA). Thermal analysis (TGA and DTA) represented in Fig. 4 were recorded for SA clay material only because of similarities. Three main losses could be noticed, the first one was at the starting of the process and was attributed to the loss of adsorbed water surface. The second loss between 428°C and 580°C which was due to the decomposition of kaolinite. The third loss which began at ~ 660 and ends at ~ 750°C was referred to the decomposition of carbonates. In DTA, four endothermic peaks and one exothermic are shown. These peaks were in agreement with the losses observed in TGA analysis. The first one, at 93°C was attributed to the loss of adsorbed water while the peak at 388°C could be attributed to the loss of structural water in the clay mineral. The third peak at 520°C, which had a high amplitude was attributed to kaolinite dehroxylation. The peak at 735°C had also a high amplitude and was attributed to the decomposition of carbonate minerals which gave rise to CO_2_ when decomposed. Finally, the peak at 928°C was attributed to phase transformation of the raw clay mineral. (Serra et al., 2013) attributed this phenomenon to pre-mullite spinal type phase formation that is not possible to detect in XRD. Similar phenomena were found when other clay minerals from different regions in Morocco were studied (Hajjaji et al., 2002; El Yakoubi, 2006; El Gamouz et al., 2007; Anbri et al., 2008; Azejjel et al., 2010; Elgamouz and Tijani, 2018, 2018a).

**Fig. 5:**
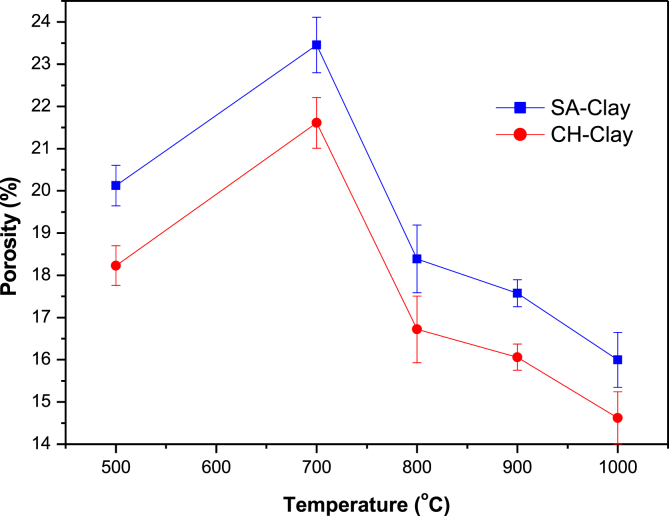
Porosity of SA and CH flat disks calculated at different final calcination temperature.

The porosity of flat disks for both samples SA and CH are represented in Fig. 5, two trends are observed, the first is an increase from initial values of 20.13% ± 0.48 and 18.23% ± 0.47 to the maximum registered values porosity of 23.45% ± 0.66 and 21.61% ± 0.60 for SA and CH clay materials respectively. These values were a direct result of capillary movements of water in the specimen pores that were opened to the outside. The increase could be explained based on the fact that, flat disks calcined at final temperature of 500°C are not consolidated enough to form a grid of macropores and mesopores which can hold water. Also, at calcination temperature of 700°C the flat disks are presented with the highest number macropores and mesopores as was demonstrated previously (Elgamouz and Tijani, 2018). An increase in final firing temperature lead to a decrease in the porosity of the flat disks down to values of 16.00% ± 0.62 and 14.62% ± 0.62 for both SA and CH clay materials. The porosity values could be used to have a good understanding of the specimen strength, especially in extreme weather conditions. A much better understanding about the strength of these materials could be gained through chemical strength which measures the resistance of the specimens to extreme pH. Chemical resistances were found to be 0.21% ± 0.08 and 0.18% ± 0.06 at pH = 5.0 and 0.23% ± 0.05 and 0.22% ± 0.05 at pH = 10 for SA and CH flat disks calcined to final temperatures of 850°C respectively. The composition of specimens was also studied after treatment with HCl or NaOH with XRD analysis, the results are included in the XRD discussion. The color of the specimen of the two clay materials were found to be prominent when specimens were fired to temperatures higher than 850°C. The prominence of the color could be explained by the oxidation of the Iron(II) oxide (FeO) to Iron(III) oxide (Fe_2_O_3_).

**Fig. 6:**
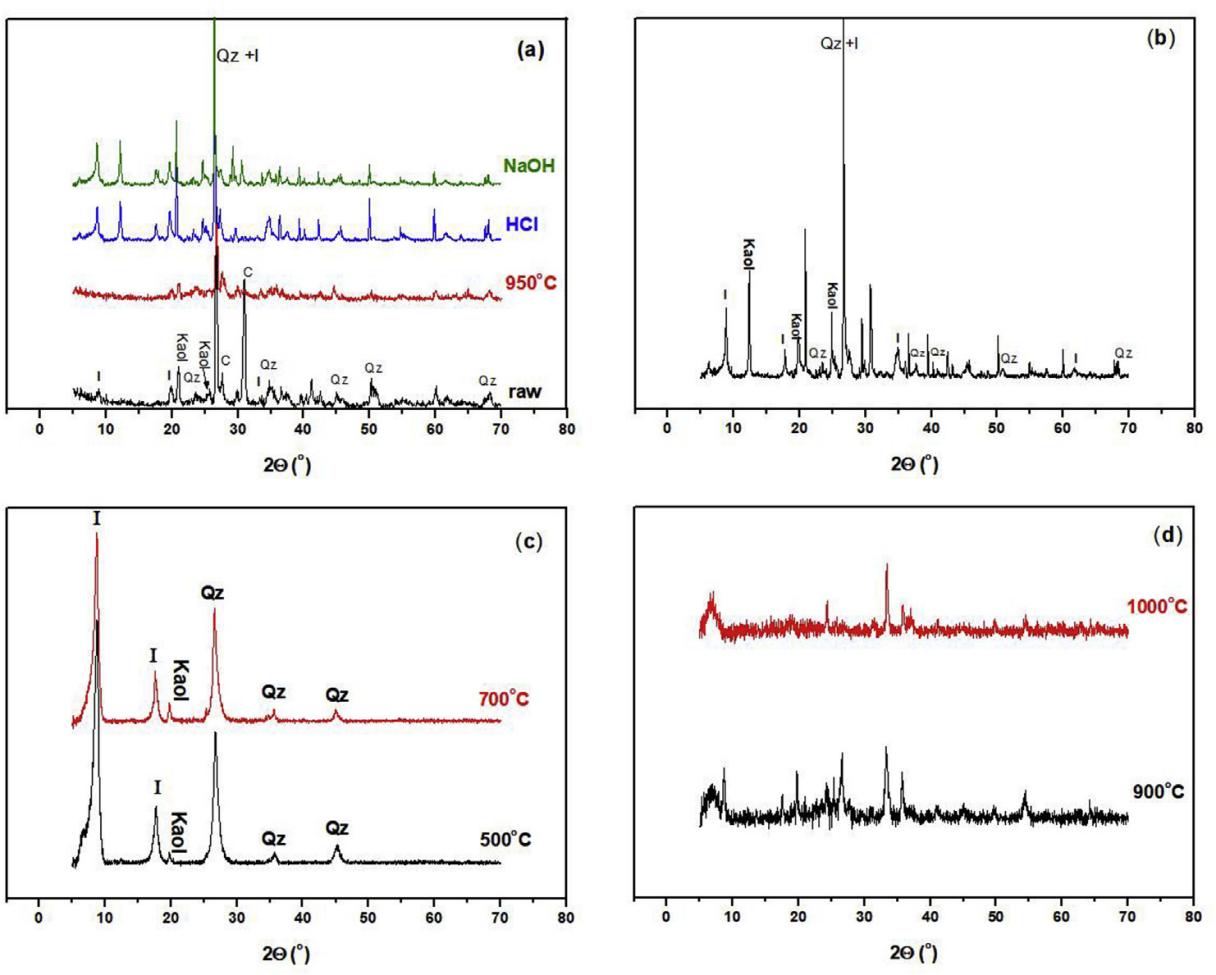
X-ray diffraction of: (a) raw, 950°C calcined, HCl and NaOH treated clay material; (b) clay fraction; (c) clay fractions calcined at 500 and 700°C and (d) calcined clay fraction at 900 and 1000°C. Abbreviations; I: illite; Kaol: kaolinite; Qz: quartz; C: calcite.

The presence of mineral oxides was investigated by XRD technique. Phyllite phases were qualitatively and quantitatively analyzed. Diffraction of the raw and calcined clay mineral at 950°C are represented in Fig. 6a. Because of similarities, only diffractions of **SA** clay mineral were presented. Quartz was the major component of the clay mineral. The interreticular measured distances, the Miller indices and the 2θ position of the diffractometric reflects exclusively of quartz are 4.27, (100), 20.9^o^; 3.35 (101), 26.7^o^; 2.45, (110), 36.5^o^; 2.12, (200), 42.5^o^ and 1.81, (112), 50.1^o^. In addition to the triplet 1.373, (212), 67.6^o^; 1.374, (203), 67.7 and 1.38, (301), 68.07^o^. Quartz diffractions were identified through powder diffraction File No 00-046-1045. It was also noted the presence of triclinic kaolinite which were characterized by interreticular distances, Miller indices and 2θ position of the diffractometric reflects, namely 7.17, (00l), 12.3^o^; 4.47, (020), 19.8^o^; 3.57, (002), 24.8^o^ and 2.38, (003), 37.9^o^ (Nakagawa et al., 2006). Kaolinite diffractions were identified through powder diffraction file No 00-014-0164. Illite was also characterized by interreticular distances, Miller indices and 2θ position of the diffractometric reflects of 10.0, (002), 8.7°; 5.02, (004), 17.6° and 3.34°, (006), 26.6°. Illite diffractions were identified through powder diffraction file No 00-026-0911. The presence of carbonate in the form of a calcite was characterized by interreticular distances, Miller indices and 2θ position of the diffractometric reflects of 3.84, (012), 23.1^o^; 3.04, (104), 29.4^o^; 2.83, (113), 39.5^o^. Calcite diffractions were identified through powder diffraction file No 00-005-0586. Calcination of the raw material to temperatures higher than 700°C leads to the disappearance of these diffractions. The clay fraction isolated from the raw material is represented in Fig. 6b. The calcination of the clay fraction to temperatures higher than 500°C leads to the kaolinite dihydroxylation; this was clearly seen in X-ray diffraction of high temperatures calcined clay fraction at 700, 900 and 1000°C represented in Figs. 6c and 6d. At such temperatures, illite was the only remaining clay fraction, the same result was found by (Serra et al., 2013) when studying the crystalline phases of the crude and a fired Argentinean calcareous commercial clay mineral. Not much difference could be assessed between specimens calcined to 950°C and those treated either with HCl or NaOH. The intense peak of carbonates at 31.15° was not present in the three samples. This is mainly due to, the loss of carbonate in the form of CO_2_ during the calcination process as explained previously. The process of carbonate decomposition may continue during HCl treatment, where carbonate is decomposed to CO_2_ and water. However, treatment of the specimen with NaOH does not favor this reaction.

The chemical composition obtained from SEM-EDAX of the two clay mineral samples are given in Table 2. Alumina, silica, iron and calcium oxides were the major constituents of the two clay mineral samples followed by magnesium and potassium oxides. Traces of titanium oxide was detected. Comparison between the composition of the raw clay mineral samples and calcined samples at 850°C showed differences in the percent of all oxides. Such observation could be explained by the phase mass loss on ignition and the transformation of Iron(II) oxide to Iron(III) oxide. The mass loss was found to be almost constant after 850°C (less than 0.26% ± 0.11) which was indicative that carbonaceous matter had already been removed at lower temperatures, contrary to the general practice of perforated brick factories where bricks are directly taken to the oven at 850°C without gradual heating program.

**Table 2:** Oxide composition of the clay samples obtained from SEM-EDAX.

Clay mineral	SiO_2_	Al_2_O_3_	Fe_2_O_3_	CaO	MgO	Na_2_O	K_2_O	TiO_2_
(CH raw)	46.98 ± 0.57	13.16 ± 0.56	4.85 ± 0.46	5.98 ± 0.33	5.87 ± 0.34	0.86 ± 0.06	2.95 ± 0.05	0.51 ± 0.01
(SA raw)	44.39 ± 0.60	19.64 ± 0.48	6.37 ± 0.70	3.34 ± 0.43	1.98 ± 0.18	0.93 ± 0.05	2.52 ± 0.03	0.60 ± 0.01
(CH calcined*)	51.64 ± 0.51	12.46 ± 0.36	3.78 ± 0.42	5.09 ± 0.14	6.34 ± 0.30	0.91 ± 0.10	3.61 ± 0.05	0.47 ± 0.01
(SA calcined*)	49.96 ± 0.61	18.65 ± 0.33	4.12 ± 0.43	3.85 ± 0.12	3.12 ± 0.32	0.82 ± 0.04	2.63 ± 0.04	0.50 ± 0.01

* Calcinations were performed at 850°C; temperature of perforated bricks at factories.

**Fig. 7:**
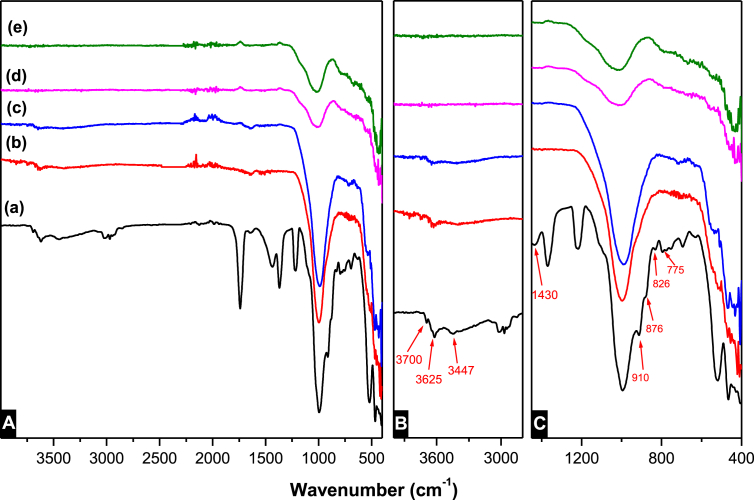
FT-IR spectra of (A) overlay spectra of (a) raw clay material, (b) clay mineral heated to 500°C, (c) heated to 700°C, (d) heated to 900°C and (e) heated to 1000°C. and (B) zoom-in of 2800–4000 cm^−1^.

Fig. 7 represents the FT-IR spectra of **SA** clay mineral. Initial inspection of the spectra revealed that the bands in the regions characteristics of clays (Nakagawa et al., 2006) (3750-3300), (3000-1800), (1450-1400), (1200-900) and (900-400) cm^−1^ were attributed to different stretching vibrations and deformation of structural hydroxides, organic fraction, carbonates, impregnated waters as well as (Si, Al, Fe)–O groups. Based on results obtained from the X-ray diffraction, the raw clay minerals belong to kaolinite and illite type. Four (-OH) vibrations were characteristic; the first three bands (3700, 3670 and 3654 cm^−1^) were attributed to the stretching vibrations of surface hydroxyl groups, while the band at 3620 cm^−1^ was attributed to inner hydroxyl vibrations of the interlayer water (Frost and Mendelovici, 2006; El Gamouz et al., 2007; Anbri et al., 2008; Elgamouz and Tijani, 2018, 2018a). Such –OH vibrational peaks disappeared due to loss of adsorbed water at high temperatures. These vibrations were manifested at lower vibration in bending modes. The (-OH) surface groups vibrated at 936 cm^−1^, while the internal bending vibration of the interlayer waters were found to vibrate at lower values of 910 cm^−1^. Vibration at 826 and 876 cm^−1^ were attributed to the stretching vibrations of metal-oxygen chemical bands, ν(Al,Fe–OH), of iron bearing kaolinite (Frost and Mendelovici, 2006). The stretching and bending vibrations of the (-OH) groups were widely used to study catalytic and adsorption properties of the kaolinite clay minerals (Djomgoue and Njopwouo, 2013; Misra et al., 2018). Presence of organic matter was also observed in the clay material even after several washings with a 10% HCl solution. Two stretching asymmetrical vibrations modes for (CH_2_) were noticed at 2970 and 2940 cm^−1^ while the lower vibration at 2860 cm^−1^ corresponded to the symmetrical vibration of the –CH_2_ group. Similar patterns were observed when a kaolinite type clay mineral was modified with 1,2-Butanediols (Murakami et al., 2004). Quartz interference was observed between 1005 and 1038 cm^−1^. The strong band at 1005 cm^−1^ was attributed to the stretching vibration of the Si–O, while the doublet at 795 and 775 cm^−1^ were the finger print of the asymmetrical and symmetrical stretching vibrations of Si–O–Si inter tetrahedral (TO_4_) bridging bonds (Saikia and Parthasarathy, 2010). Among various vibration of the carbonate, the frequency at 1430 cm^−1^ was attributed to ${\text{CO}}_{3}^{2-}$ stretching vibration, while frequencies of 1725 and 1740 cm^−1^ are attributed to C=O stretching mode. The vibrations of carbonates disappeared when the clay material was heated above 500°C (Senthil Kumar and Rajkumar, 2013).

**Fig. 8:**
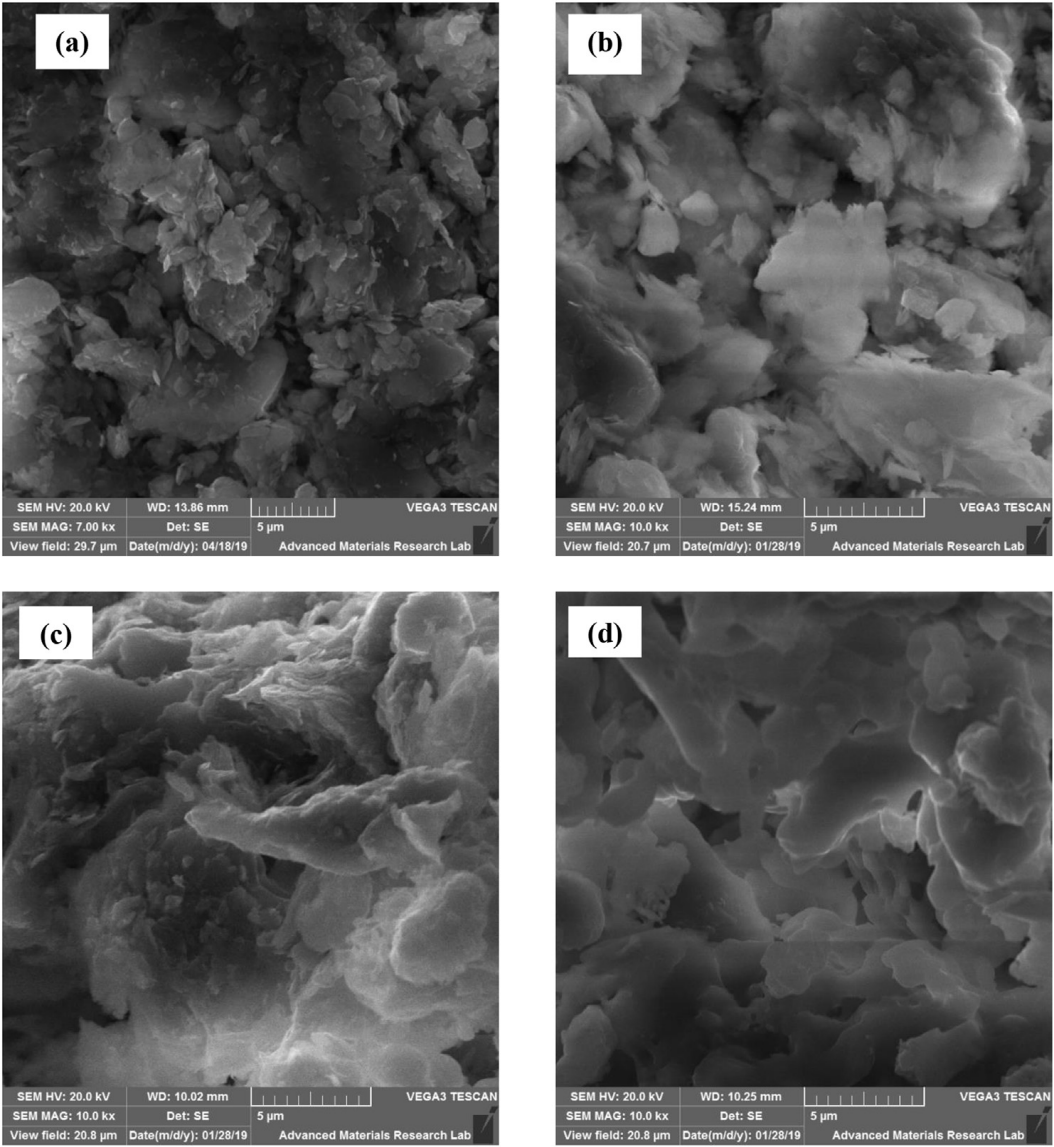
SEM micrographs of the clay material heated to different temperature. (a) raw, (b) T = 550°C; (c) T = 850°C; (d) glassy phase T = 1000°C.

Figs. 8(a), 8(b), 8(c) and 8(d) show the SEM of the clay mineral which were the morphology (configuration) of a spray-dried powder. Fig. 8(a) indicated that the clay mineral was constituted from fine poorly crystalline particles with most of the size distribution of more than 5μm. The small platelets in Fig. 8(b) indicated the presence of crystalline phases. While Fig. 8(c) illustrated the starting of a glassy phase where clay particles started fusing with each other under the effect of heat to give a ceramic material with very low porosity. Fig. 7(d) is an advance stage of the glassy phenomenon where clay granules fused into each other and became aggregates.

Results of blue value (V_B_) and blue index (MIB) are represented in Table 3. The blue values (V_B_) for both samples of the clay material and bentonite were obtained from the tests performed according to the AFNOR method were always higher than those obtained from the tests performed according to the ASTM procedure. Also, the blue value (V_B_) and blue index (MIB) of the bentonite were higher than the ones for the clay mineral samples which revealed the poorness of the clay mineral sample from phyllite phases and the purity of the bentonite. Hence, bentonite was used as reference in this study.

**Table 3:** Blue values (V_B_) and blue indices (MIB) found from different tests.

V_B_ (g/100g)					MIB (10^−3^ eq/100g)	
	AFNOR				ASTM	
Sample	a pH not controlled	b pH not controlled	c pH controlled	d pH controlled	a pH not controlled	e pH controlled
Clay mineral	6.6 ± 0.2	5.0 ± 0.1	4.5 ± 0.3	5.3 ± 0.4	13.5 ± 0.5	4.5 ± 0.3
Bentonite	19.0 ± 0.1	19.5 ± 0.3	19.5 ± 0.2	17.7 ± 0.3	50.0 ± 0.1	16.0 ± 0.5

a [MB] = 10 g/L; Solid/Liquid = 0.6 g/L.

b [MB] = 10 g/L; Solid/Liquid = $6.7\times 10^{-3}\;g/L$.

c [MB] = 10 g/L; Solid/Liquid = $6.7\times 10^{-3}\;g/L$.

d [MB] = 3.2 g/L; Solid/Liquid = $6.7\times 10^{-3}\;g/L$.

e [MB] = 3.2 g/L; Solid/Liquid = $6.7\times 10^{-3}\;g/L$.

Comparison between the blue values obtained at different conditions, with and without pH control, revealed the influence of the environmental control. Initial pH values were basic in the range of 9.0 ± 0.4. A decrease in the pH to values of 2.5 ± 0.3 of the initial solution lead to a decrease in the blue value (V_B_). The surface charge of the clay mineral was affected by the pH of the solution. Low acidic pH lead to a less basic surface and therefore to a less methylene blue uptake by the clay mineral. Methylene blue is a cationic dye with excessive positive charge in solution lead to competition between methylene blue and positive charges provided by the acidic interaction sites on the clay material which produced faded blue stains. Similar results were reported for the adsorption of methylene blue onto clay materials (Auta and Hameed, 2012; Cherifi et al., 2013; Youssef et al., 2014).

The capacity of a soil to adsorb methylene blue is directly related to concentration of clay fractions in the soil. The amount of adsorbed methylene blue can be used to determine the specific surface area S_a_, in m^2^/g of the tested clay minerals and the reference bentonite. The blue value calculated using AFNOR or ASTM assays represents the capacity of a soil to uptake methylene blue in (g of MB/100 g of material). Knowing that, 0.01 g/mL is the concentration of MB used in the titration, and its monovalent flat-lying structure surface area of 132 Å^2^ (Patel and Vashi, 2015), VB could be converted to a surface by multiplying by Avogadro's number and the surface area of MB and dividing by the molar mass of MB. Eq. (7) was obtained from this conversion and was used to calculate the specific surface area of the clay materials.

(7) Sa=(VB100)×(NMMMB)×(132×10−20)=21VB

In Eq. (7), N is Avogadro's number (6.022 × 10^23^), and MM_MB_ is the molar mass of the methylene blue (373.0 g mol^−1^), the surface of MB was converted from 132 Å^2^ to $132\times 10^{-20}$ m^2^. The specific surface areas were obtained from different V_B_ values are represented in Table 4.

**Table 4:** Specific surface area calculated from different V_B_ values at different conditions specified previously in Table 3.

	Conditions	V_B_ (g/100g)	S_a_ (m^2^/g)
SA clay mineral AFNOR	apH not controlled	6.6 ± 0.2	138.6 ± 4.2
	bpH not controlled	5.0 ± 0.1	105 ± 2.1
	cpH controlled	4.5 ± 0.3	94.5 ± 6.3
	dpH controlled	5.3 ± 0.4	111.3 ± 4.2
Bentonite AFNOR	apH not controlled	19.0 ± 0.1	399 ± 2.6
	bpH not controlled	19.5 ± 0.3	409.5 ± 6.3
	cpH controlled	19.5 ± 0.2	409.5 ± 4.2
	dpH controlled	17.7 ± 0.3	371.7 ± 6.3
SA clay mineral ASTM	apH not controlled	13.5 ± 0.5	93.8 ± 10.5
	epH controlled	4.5 ± 0.3	--
Bentonite ASTM	apH not controlled	50.0 ± 0.1	1,050.0 ± 2.1
	epH controlled	16.0 ± 0.5	366.0 ± 10.5
Reference materials	inert minerals	--	4–20
	chlorite, illite, kaolinite	--	10–100
	smectite & vermiculite	--	100–700

a [MB] = 10g/L; Solid/Liquid = 0.6 g/L.

b [MB] = 10g/L; Solid/Liquid = $6.7\;\times \;\frac{10^{-3}g}{L}$.

c [MB] = 10g/L; Solid/Liquid = $6.7\;\times \;\frac{10^{-3}g}{L}$.

d [MB] = 3.2 g/L; Solid/Liquid = $6.7\;\times \;\frac{10^{-3}g}{L}$.

e [MB] = 3.2 g/L; Solid/Liquid = $6.7\;\times \;\frac{10^{-3}g}{L}$.

The total active surface was found to vary between 93.8 ± 10.5 to 138.6 ± 4.2 m^2^/g for the clay mineral. While the average specific surface area of bentonite was found to be equal to 500.7 ± 13.6 m^2^/g using methylene blue test, which is in accordance with values between 462 to 787 ± 10.0 m^2^/g found by El Miz et al. for a Moroccan bentonite taken from the northern region (Nador) using the ethylene glycol method (El Miz et al., 2017). This was indicative that the clay material studied in this work falls within the chlorite, illite, and kaolinite categories. Results are in agreement with the X-ray diffraction which showed that the clay mineral was composed mainly from kaolinite, illite and carbonate. Previous studies about clay minerals from Morocco showed that they all belong to the previously mentioned categories (Karim et al., 2009; Hajjaji and Mezouari, 2011; Ainane et al., 2014; El Ouahabi et al., 2014). Biron's studies supported that clay minerals in the Atlas Mountains belonged to the 6^th^ uppermost unit of six successive geological parts which were developed during the Permian and Triassic ages (Biron, 1982; Biron and Courtinat, 1982).

## 4 Conclusion

In this paper, an abundantly available natural clay mineral was successfully characterized by different physicochemical techniques. Findings of this study suggest, that the clay minerals from Safi region are mainly constituted from quartz with a mass% ranging from 44.40% ± 0.60–46.98% ± 0.57. The pure clay fractions of the clay minerals were illite and kaolinite, these appear to have a direct impact on the surface area of the clay mineral which was demonstrated through methylene blue stains test. The final characteristics such as shrinking and porosity of the specimens made from the clay minerals were found to be directly affected by the thermal phenomena that happened during the heating process. The importance of this work lies in providing an insight on how methylene blue test could be used to quantify pure clay contents of a common clay mineral. Nevertheless, this test may suffer from limitation because of the swelling of the material. The present findings might help to pre-characterize exploited depot clay minerals that are widely used for the fabrication of ceramic products such as clay kitchenware, perforated bricks and ceramic membranes supports. Future work will concentrate on the fabrication of tubular porous ceramic membranes, porogens such as activated carbon and starch will be used to enhance porosity in the clay supports.

## Declarations

### Author contribution statement

Abdelaziz Elgamouz: Conceived and designed the experiments; Performed the experiments; Analyzed and interpreted the data; Contributed reagents, materials, analysis tools or data; Wrote the paper.

Najib Tijani: Conceived and designed the experiments; Contributed reagents, materials, analysis tools or data; Wrote the paper.

Ihsan Shehadi, Kamrul Hasan: Contributed reagents, materials, analysis tools or data; Wrote the paper.

Mohamed Kawam: Performed the experiments; Contributed reagents, materials, analysis tools or data.

### Funding statement

This work was supported by the Research Institute of Science and Engineering (RISE) [grant numbers 1602142018, 2016].

### Competing interest statement

The authors declare no conflict of interest.

### Additional information

No additional information is available for this paper.

## Appendix A Supplementary data

Data_in_brief_Helyion Data_in_brief_Helyion
