# Erratum to “Characterization of the firing behaviour of an illite-kaolinite clay mineral and its potential use as membrane support” [Heliyon 5, (8), (August 2019), e02281]

**DOI:** 10.1016/j.heliyon.2019.e02714

**Published:** 2019-12-06

**Authors:** Abdelaziz Elgamouz, Najib Tijani, Ihsan Shehadi, Kamrul Hasan, Mohamad Al-Farooq Kawam

**Affiliations:** a Department of Chemistry, College of Sciences, Research Institute of Science and Engineering, University of Sharjah, P.O. Box 27272, Sharjah, United Arab Emirates; b Equipe Membranes, Matériaux et Procédés de Séparation, Faculté des Sciences, Université Moulay Ismaîl, Meknes, Morocco

In the original published version of this article, the publisher accidentally published a file as a supplement. There should not have been any supplementary file. The publisher apologises for the error. Both the HTML and PDF versions of the article have been updated to correct the error.

