# Supplementary material for: Characterization of the firing behaviour of an illite-kaolinite clay mineral and its potential use as membrane support
Source: Heliyon. 2019 Aug 30;5(8):e02281. doi: 10.1016/j.heliyon.2019.e02281 (PMC6726589; doi:10.1016/j.heliyon.2019.e02281)
Supplement: Data_in_brief_Helyion [file mmc1.docx]

**Dataset for the characterization of an illite-kaolinite clay mineral**

**Authors**: **Abdelaziz Elgamouz^*a^, Najib Tijani^b^, Ihsan Shehadi^a^, Kamrul Hasan^a^** and **Mohamad Al-Farooq Kawam^a^**

**Affiliations**:

^a^Department of Chemistry, College of Sciences, Research Institute of Science and Engineering, University of Sharjah, P.O. Box 27272, Sharjah, United Arab Emirates

^b^Group : Membranes, Matériaux et Procédés de Séparation, Faculté des Sciences,

Université Moulay Ismaîl, Meknès, Morocco

**Contact email**: *Email:* [*aelgamouz@sharjah.ac.ae*](mailto:aelgamouz@sharjah.ac.ae)

**Abstract**

This article describes the data generated from multiple approach methodology physico-chemical characterization of a clay mineral from the West-Central region of Morocco, Safi province. Data were generated from classical chemical analytical techniques namely: organic matter content, dilatometric analysis, weight loss on ignition, porosity and methylene blue stain tests according to the Association Française de Normalization (AFNOR) and American Society for Testing and Materials (ASTM). In addition to data generated using instrumental analytical techniques namely: Infrared spectroscopy (FTIR), thermal gravimetric analysis (TGA) and deferential thermal analysis (DTA), X-ray diffraction (XRD), scanning electron microscopy (SEM) and elemental energy disperse spectroscopy (EDX).

**Specifications Table**

| Subject area | *chemistry* |
| --- | --- |
| More specific subject area | *Clays* |
| Type of data | Table, image, text file, graph, figure |
| How data was acquired | - ARL-8660 X-Ray Fluorescence Spectrometer   SETARAM TGA instrument   - Bruker Platinum ATR tensor II FTIR spectrometer - NABER 2804 furnace - X-*ray* diffraction (XRD) using a D-Max Rigaku X-ray diffractometer with a copper anode and a graphite monochromator to select CuK_α1_ radiation (λ = 1.540 Å). - *Tescan* VEGA XM variable pressure SEM equipped with Oxford Instruments X-Max 50 EDX detector, controlled with AZtecEnergy analysis software with a resolution of 125 eV to determine the abundance of elements. |
| Data format | Crude, filtered, analyzed |
| Experimental factors | The clay material was crushed to coarse material, then to fine powder followed by the sieving operation using standardized AFNOR sieves in the range 250-315 µm. |
| Experimental features | The clays were characterized by various methods: XRD, SEM-EDX, FTIR, TGA/TDA and later semi-quantitatively found the concentration of the pure clay fractions (illite and kaolinite) using the blue stain test according to AFNOR and ASTM. |
| Data source location | The clay material used in this study was collected from Safi region which is adjacent to Middle Atlas. The site of sampling is situated at about 10 Km from Safi center. (geographical coordinates: Latitude: 32° 16' 60.00" North and the Longitude: -9° 13' 60.00" West). |
| Data accessibility | The data represented is with this article. |
| Related research article | This data article is submitted as a companion paper to the research article entitled: “Characterization of a Safi Illite-Kaolinite clay by means of multiple-technique methodology with special focus on methylene blue stain test”, by A. Elgamouz, *et al.*, to be published in the Journal of Applied Clay Science [1]. |

**Value of the Data**

- The data presents very useful results for the characterization of clays.
- This data gives a detailed and complete set of experiments on the characterization of the clay fraction in a given soil.
- The data would allow other researchers to identify the key parameters that need to be controlled when investigating a new clay material.

1. **Data**

Obtaining good ceramic products without deformation or cracks is related to the properties owned by a clay mineral when water is added to it. The amount of water that can be up-taken by the clay affect the final characteristics of the final ceramic specimen. Data on quantity of water absorbed, loss on ignition, drying characteristics, dilatation and shrinking, apparent density and chemical resistance of the specimens are given in the table below.

- 1. **Equations used in classical method analysis**

***1.1.1 Weight loss on ignition***

| ** | **Eq. 1** |
| --- | --- |

In Eq.1, m_110_ is the specimen’s weight at 110 °C, m_T_ is the specimen’s weight fired at final temperature of 250, 500, 700, 800, 850, 900, 950, 1000, 1050 and 1100^o^C.

***1.1.2 Shrinkage analysis***

| ** | **Eq.2** |
| --- | --- |

In Eq. 2, L_0_ is the length between the two marks recorded on specimen before firing and L_T_ is the length between the two marks recorded on the specimen after the calcination at final temperature T.

***1.1.3 Water absorption and porosity***

| *$\text{P \% }=\left(\frac{m_{f}-m_{0}}{m_{0}}\right)\times100$* | **Eq.3** |
| --- | --- |

In Eq.3, m_0_ is the initial weight and m_f_ is the finale weight of the specimen after firing.

***1.1.6 Apparent density***

The apparent solid density, abbreviated as apparent density, is the ratio of the mass of the clay specimen to apparent volume of the specimen calculated by closing the open pores of the specimen by wax.

| $D_{A}=\frac{m}{V_{o}-V_{C}}$ | **Eq. 4** |
| --- | --- |

In Eq. 4, m is the mass of the clay specimen fired to 850 ^o^C, and Vo is its volume with the open pores. V_C_ is its volume after closing the pores using wax.

***1.1.7 Chemical resistance***

| $R=\frac{m_{o}-m_{pH,10}}{m_{o}}$ | **Eq. 5** |
| --- | --- |

In Eq. 5 m_o_ is the mass of the specimen before pH attack and mpH, is its mass after removing the specimen from the acidic (HCl, pH = 5.0) or basic (NaOH, pH =10.0) solutions for 24 hours

Data obtained using classical analytical techniques are represented in Table 1.

**Table. 1** Safi clay characteristics studied preliminarily before the physico-chemical characterization.

| **Clay sample** | **SA** | **CH** |
| --- | --- | --- |
| quantity of water absorbed  mL/Kg | 300 | 200 |
| Loss at 500 ^o^C | 0.91 | 1.81 |
| loss on ignition  850 °C | 11.47 | 11.03 |
| drying characteristics Shrinking | 1.45 % | 1.38 % |
| apparent density (g/cm3) | 0.61 | 0.26 |
| chemical resistance at pH = 5.0 | 0.21 | 0.25 |
| chemical resistance at pH = 10.0 | 0.23 | 0.30 |

- 1. **Loss on Ignition determination for SA clay sample**

| $LOI\%=\left( \frac{m_{d}-m_{c}}{m_{d}} \right)\times100$ | **Eq. 1** |
| --- | --- |

Where, $m_{d}$ is the mass of dry clay sample and $m_{c}$is mass of calcined clay sample. Results for loss on ignition for SA clay sample are given in Tables 2 to 4.

**Table. 2** Safi clay calcined to 850°C (at rate of 5°C/min) for 1hr.

| **Crucible No.** | **Dry clay mass** | **Calcined clay mass** | **LOI%** |
| --- | --- | --- | --- |
| **1** | 1.0018 g | 0.8834 g | 11.8187 % |
| **2** | 1.0002 g | 0.8843 g | 11.5877 % |
| **3** | 1.0197 g | 0.9074 g | 11.0130 % |

**Table.3** Safi clay calcined to 1000°C (at rate of 5°C/min till 550°C then at rate of 8°C/min) for 1hr.

| **Crucible No.** | **Dry clay mass** | **Calcined clay mass** | **LOI%** |
| --- | --- | --- | --- |
| **1** | 1.0018 g | 0.8792 g | 12.2380 % |
| **2** | 1.0002 g | 0.8790 g | 12.1176 % |
| **3** | 1.0197 g | 0.9031 g | 11.4347 % |

**Table.4** Safi clay LOI from 850°C to 1000°C

| **Crucible No.** | **Dry clay mass** | **Calcined clay mass** | **LOI%** |
| --- | --- | --- | --- |
| **1** | 0.8834 g | 0.8792 g | 0.4754 % |
| **2** | 0.8843 g | 0.8790 g | 0.5993 % |
| **3** | 0.9074 g | 0.9031 g | 0.4739 % |

**1.2 Chemical composition of the clay**

The elemental chemical analysis of the natural clay was performed by a *Tescan* VEGA-XM SEM spectrometer equipped with Oxford Instruments X-Max 50 EDX detector. This analysis shows that the clay samples consist mainly of silicon oxide SiO_2_ (44.40 ± 0.60 to 46.98 ± 0.57 m%), aluminium oxide (13.16 ± 0.56 to 19.64 ± 0.48 m%), iron oxide (4.85 ± 0.46 to 6.37 ± 0.70 m %), and relatively smaller amounts of alkaline-earth oxides (3.34 ± 0.43 to 5.98 ± 0.33 m% calcium oxide and 1.98 ± 0.18 to 5.87 ± 0.34 m% for magnesium oxide), data is represented in Table 1.

**1.3 Oxides compositions of the clay**

The EDX measurements were acquired from the characteristic peaks of elements present in the clay (Na, K, Mg, Ca, Al, Si, S, Ti and Fe). Elements concentrations were determined after treatment of signals and the main outcome of the analysis is the (K-ratio). The concentration of the i-th element in the sample was calculated using Eq. 2.[2,3].

| $\frac{C_{i}}{C_{(i)}}=ZAF\times\frac{I_{i}}{I_{(i)}}=ZAF\times K-ratio$ | **Eq. 2** |
| --- | --- |

The oxides concentration was calculated by using the "Elements by difference" in advanced pan EDX software, oxygen concentration was calculated as difference between 100% and the sum of all other elements. Then, percentages of oxides were calculated by combining oxygen with all other elements that can form oxides [4]. Based on previous studies on clay oxides used were: Na_2_O, MgO, Al_2_O3, SiO_2_, SO_3_, Cl_2_O, K_2_O, CaO, TiO_2_ and Fe_2_O_3_ [5,6]. The compositions of the two clay samples SA and CH in oxides at different temperatures are given in Tables 5 to 10.

**Table 5**: Percentages of the oxides composing the SA clay crude.

| Elem | Wt % | Mol % | K-Ratio | Z | A | F |
| --- | --- | --- | --- | --- | --- | --- |
| Na_2_O | 1.42 | 1.6 | 0.0028 | 0.967 | 0.2776 | 1.0046 |
| MgO | 4.48 | 7.76 | 0.0107 | 0.992 | 0.3954 | 1.0084 |
| Al_2_O_3_ | 25.24 | 17.3 | 0.0662 | 0.9634 | 0.5101 | 1.009 |
| SiO_2_ | 51.88 | 60.33 | 0.1209 | 0.992 | 0.5021 | 1.0013 |
| SO_3_ | 1.37 | 1.19 | 0.0029 | 0.9851 | 0.5436 | 1.0031 |
| Cl_2_O | 0.49 | 0.39 | 0.0024 | 0.9364 | 0.6477 | 1.0048 |
| K_2_O | 4.54 | 3.36 | 0.029 | 0.9404 | 0.8145 | 1.0057 |
| CaO | 3.7 | 4.61 | 0.0216 | 0.964 | 0.8458 | 1.0029 |
| TiO_2_ | 0.99 | 0.87 | 0.0048 | 0.8839 | 0.9158 | 1.0053 |
| Fe_2_O_3_ | 5.9 | 2.58 | 0.0361 | 0.8841 | 0.99 | 1.0 |
| Total | 100 | 100 |  |  |  |  |

**Table 6**: Percentages of the oxides composing the SA clay calcined to 850 ^o^C.

| Elem | Wt % | Mol % | K-Ratio | Z | A | F |
| --- | --- | --- | --- | --- | --- | --- |
| Na_2_O | 0.83 | 2.04 | 0.0034 | 0.9703 | 0.2589 | 1.0042 |
| MgO | 4 | 12.34 | 0.0157 | 0.9953 | 0.3674 | 1.0071 |
| Al_2_O_3_ | 22.37 | 15.46 | 0.0534 | 0.9666 | 0.4632 | 1.0079 |
| SiO_2_ | 48.61 | 57.02 | 0.1092 | 0.9953 | 0.4823 | 1.001 |
| SO_3_ | 1.37 | 1.19 | 0.0029 | 0.9851 | 0.5436 | 1.0031 |
| Cl_2_O | 0.49 | 0.39 | 0.0024 | 0.9364 | 0.6477 | 1.0048 |
| K_2_O | 4.32 | 3.23 | 0.0279 | 0.9441 | 0.82 | 1.0066 |
| CaO | 3.29 | 4.14 | 0.0195 | 0.9676 | 0.8516 | 1.0049 |
| TiO_2_ | 0.5 | 0.44 | 0.0025 | 0.8871 | 0.9212 | 1.0112 |
| Fe_2_O_3_ | 4.12 | 5.32 | 0.0742 | 0.8874 | 0.993 | 1.0 |
| Total | 100 | 100 |  |  |  |  |

**Table 7**: Percentages of the oxides composing the SA clay calcined to 950 ^o^C.

| Elem | Wt % | Mol % | K-Ratio | Z | A | F |
| --- | --- | --- | --- | --- | --- | --- |
| Na_2_O | 0.82 | 0.73 | 0.0015 | 0.9521 | 0.2607 | 1.0027 |
| MgO | 2.7 | 3.71 | 0.006 | 0.9766 | 0.3783 | 1.0049 |
| Al_2_O_3_ | 18.6 | 7.87 | 0.037 | 0.9485 | 0.506 | 1.0055 |
| SiO_2_ | 49.96 | 26.74 | 0.0739 | 0.9768 | 0.5575 | 1.0007 |
| SO_3_ | 1.37 | 1.19 | 0.0029 | 0.9851 | 0.5436 | 1.0031 |
| Cl_2_O | 0.49 | 0.39 | 0.0024 | 0.9364 | 0.6477 | 1.0048 |
| K_2_O | 2.63 | 1.55 | 0.0182 | 0.9236 | 0.9004 | 1.0048 |
| CaO | 2.44 | 2.41 | 0.0153 | 0.9473 | 0.925 | 1.0022 |
| TiO_2_ | 0.5 | 0.35 | 0.0025 | 0.8691 | 0.9706 | 1.0042 |
| Fe_2_O_3_ | 3.37 | 1.17 | 0.0207 | 0.869 | 1.0123 | 1.0 |
| Total | 100 | 100 |  |  |  |  |

**Table 8**: Percentages of the oxides composing the crude CH clay.

| Elem | Wt % | Mol % | K-Ratio | Z | A | F |
| --- | --- | --- | --- | --- | --- | --- |
| Na_2_O | 0.96 | 0.85 | 0.0017 | 0.9514 | 0.2552 | 1.0024 |
| MgO | 6.34 | 2.97 | 0.0048 | 0.976 | 0.3699 | 1.0043 |
| Al_2_O_3_ | 12.92 | 6.94 | 0.0326 | 0.9479 | 0.5 | 1.0049 |
| SiO_2_ | 25.44 | 23.18 | 0.0653 | 0.9762 | 0.5619 | 1.0008 |
| SO_3_ | 0.46 | 0.31 | 0.0012 | 0.9695 | 0.6787 | 1.0023 |
| K_2_O | 2.66 | 1.55 | 0.0187 | 0.9229 | 0.9102 | 1.0052 |
| CaO | 2.42 | 2.36 | 0.0153 | 0.9466 | 0.9324 | 1.0026 |
| TiO_2_ | 0.5 | 0.33 | 0.0024 | 0.8685 | 0.9752 | 1.0053 |
| Fe_2_O_3_ | 4.15 | 1.42 | 0.0255 | 0.8684 | 1.014 | 1 |
| Total | 100 | 100 |  |  |  |  |

**Table 9**: Percentages of the oxides composing the CH clay calcined to 850 ^o^C.

| Elem | Wt % | Mol % | K-Ratio | Z | A | F |
| --- | --- | --- | --- | --- | --- | --- |
| Na_2_O | 0.85 | 0.73 | 0.0016 | 0.9514 | 0.2672 | 1.003 |
| MgO | 7.09 | 9.41 | 0.0162 | 0.9759 | 0.3871 | 1.0046 |
| Al_2_O_3_ | 10.29 | 5.4 | 0.0252 | 0.9478 | 0.4857 | 1.0059 |
| SiO_2_ | 31.11 | 27.71 | 0.0802 | 0.9761 | 0.5647 | 1.0009 |
| SO_3_ | 0.47 | 0.31 | 0.0012 | 0.9694 | 0.6566 | 1.0023 |
| K_2_O | 2.76 | 1.57 | 0.019 | 0.9229 | 0.8966 | 1.0051 |
| CaO | 3.01 | 2.87 | 0.0188 | 0.9466 | 0.9212 | 1.0015 |
| TiO_2_ | 0.47 | 0.31 | 0.0024 | 0.8685 | 0.966 | 1.0025 |
| Fe_2_O_3_ | 2.03 | 0.68 | 0.0125 | 0.8683 | 1.0105 | 1 |
| Total | 100 | 100 |  |  |  |  |

**Table 10**: Percentages of the oxides composing the CH clay calcined to 950 ^o^C.

| Elem | Wt % | Mol % | K-Ratio | Z | A | F |
| --- | --- | --- | --- | --- | --- | --- |
| Na_2_O | 0.91 | 0.82 | 0.0017 | 0.9562 | 0.2646 | 1.0033 |
| MgO | 9 | 12.54 | 0.0205 | 0.9809 | 0.3824 | 1.0049 |
| Al_2_O_3_ | 11.6 | 6.39 | 0.0276 | 0.9526 | 0.4684 | 1.0064 |
| SiO_2_ | 34.19 | 31.96 | 0.085 | 0.981 | 0.5413 | 1.0012 |
| SO_3_ | 0.55 | 0.38 | 0.0013 | 0.9742 | 0.6294 | 1.0032 |
| K_2_O | 3.61 | 2.15 | 0.0246 | 0.9284 | 0.879 | 1.0077 |
| CaO | 5.09 | 5.1 | 0.0313 | 0.952 | 0.902 | 1.0019 |
| TiO_2_ | 0.47 | 0.31 | 0.0024 | 0.8685 | 0.966 | 1.0025 |
| Fe_2_O_3_ | 4.43 | 1.56 | 0.0271 | 0.8733 | 1.0032 | 1 |
| Total | 100 | 100 |  |  |  |  |

**1.4 X-ray diffraction**.

The clay powder was analyzed using XRD, it was revealed presence of a strong peak of silica in the form of quartz, calcite, and illite and kaolinite as the only pure clay fractions. Figure 1 and 2 represents the X-ray diffraction of two clay sample studied, the spectra of the two clay samples are almost identical.

**Figure-1** X-ray diffraction of the crude SA clay sample. Q: Quartz, C: Calcite, I: Illite and K: Kaolinite

**Figure-1** X-ray diffraction of the crude CH clay sample. Q: Quartz, C: Calcite, I: Illite and K: Kaolinite

**1.5 Infrared spectroscopy.**

The FTIR analysis was carried out in the spectral range (400-4000) cm^-1^ by a Bruker Platinum ATR tensor II spectrometer with a resolution of 4 cm^-1^. Figure 2 represents the FTIR spectrum of natural clay.

| 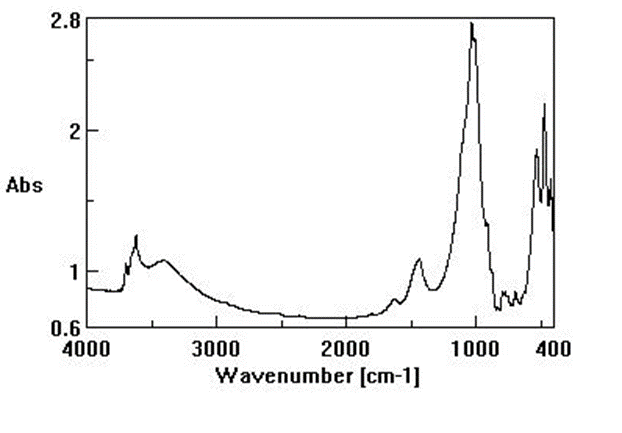  H_2_O Bend  Carbonates  **OH inter- layer water**  **OH Surface water**  (**a**) | 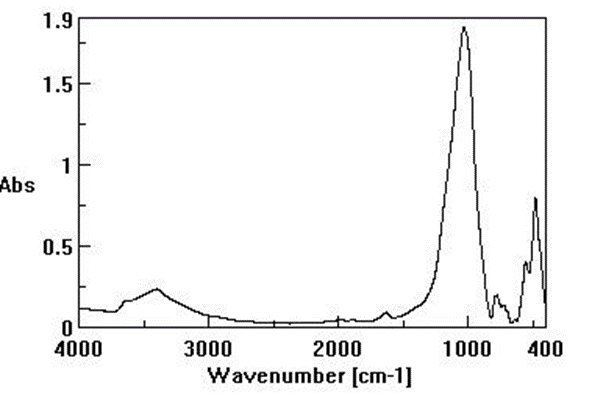  Carbonates  (**b**)  **SiO_2_** |
| --- | --- |
| 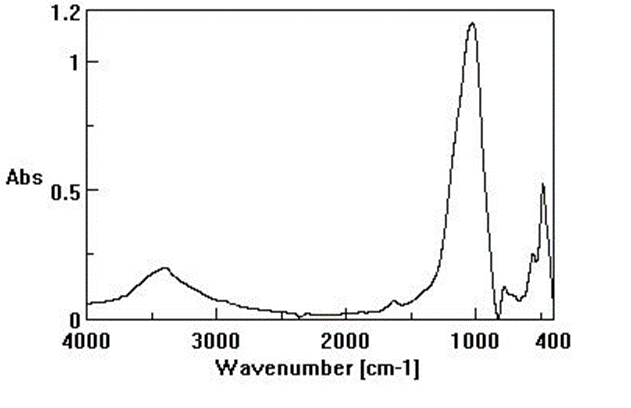  **SiO_2_**  (**c**) | 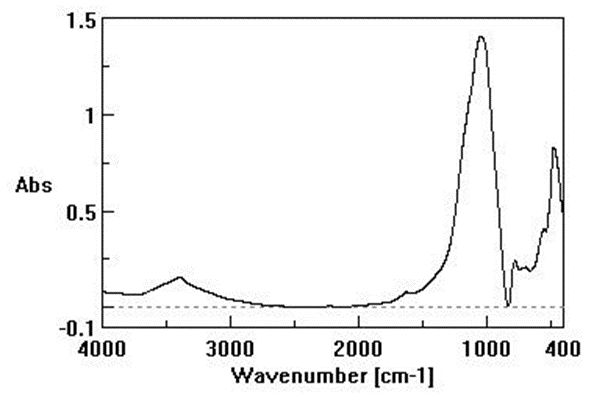  **SiO_2_**  (**d**) |

**Figure 3** FTIR spectrum of the SA natural clay sample at different temperatures showing the main vibrations, (a) crude material, (b) calcined to 250 ^o^C, (c) calcined to 500 ^o^C and (d) calcined to 850 ^o^C.

| 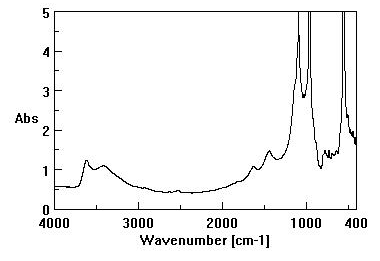  H_2_O Bend  Carbonates  **OH inter- layer water**  **OH Surface water**  (**a**) | 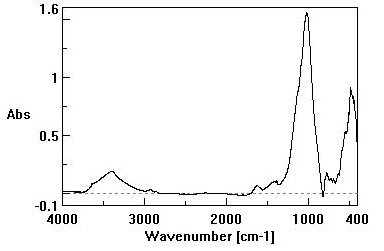  (**b**)  **SiO_2_** |
| --- | --- |
| 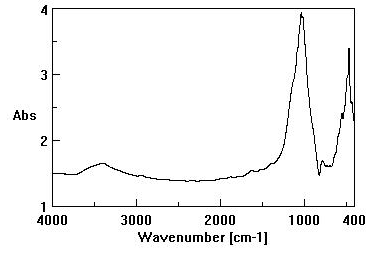  (**c**)  **SiO_2_** | 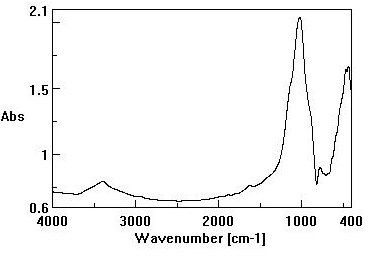  (**d**)  **SiO_2_** |

**Figure 11** FTIR spectrum of the CH natural clay sample at different temperatures showing the main vibrations, (a) crude material, (b) calcined to 250 ^o^C, (c) calcined to 500 ^o^C and (d) calcined to 850 ^o^C.

**Table 2.** Infrared peak attributions of the SA and CH clay minerals at different temperatures.

| **IR Frequencies of crude clay** | **IR Frequencies of calcined clay to 850 ^o^C** | **Attributions** |
| --- | --- | --- |
| 3708 | **Disappeared because of calcination** | ν Si-OH external (SiO_2_) |
| 3632 | 3660 | ν Al-OH external (Al_2_O_3_) |
| 3406 | 3412 | ν OH (H_2_O) interlayer water |
| 1625 | 1640 | δ OH(OH2) [7] |
| 1437 | **Disappeared because of calcination** | ν CO3 |
| 1031 | 1034 | ν Si-O (SiO_2_) |
| 909 | **Disappeared because of calcination** | δ(Al.Si.Mg.Ca)OH |
| 870 | **Disappeared because of calcination** | δ(CO3) |
| 786 | **Disappeared because of calcination** | δ(CO3) |

ν: stretching vibration, δ : bending vibration.

**1.6 Thermal analysis**.

The thermogravimetric (TGA) and differential thermal analysis were carried out, they show similarities. The graph of analysis is given in Figure 5, and various phenomena attributions are given in Table 3.

**Figure 5** Thermal analysis (TGA and DTA) of the SA clay crude sample.

**Table 12.** Thermal analysis (TGA/TDA) attribution of different phenomena of the SA and CH clay samples.

| **TGA weight losses' intervals (^o^C)** | **TDA Peaks** | **Phenomenon** | **Attribution** |
| --- | --- | --- | --- |
| 30-123 | 90 | endothermic | departure of the water adsorbed on surface of the clay |
| 366-402 | 390 | endothermic | Interlayer water departure |
| 465-587 | 530 | endothermic | Kaolinite and illite decompositions |
| 674-760 | 740 | endothermic | Calcite decomposition |
| 908-935 | 925 | exothermic | Mullite crystallization |

**1.7 SEM analysis**

| 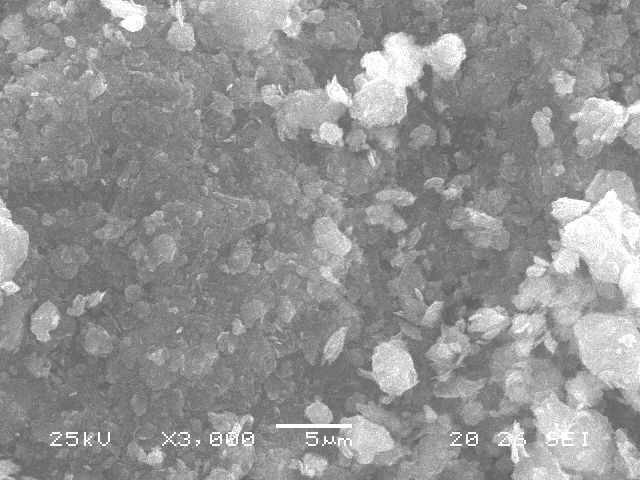  **(a)** | 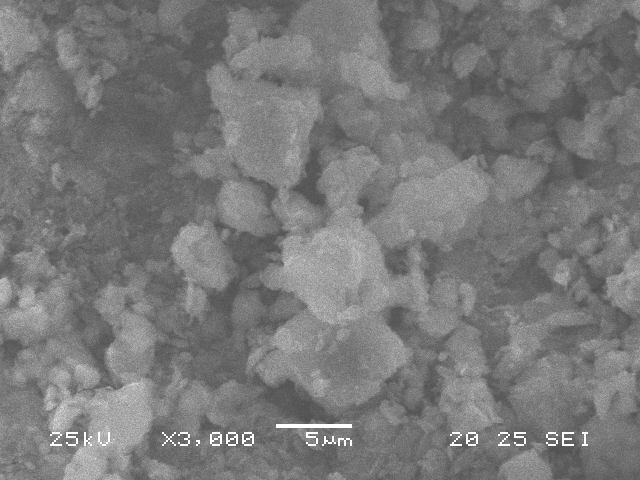  **(b)**  **(d)** |
| --- | --- |
| 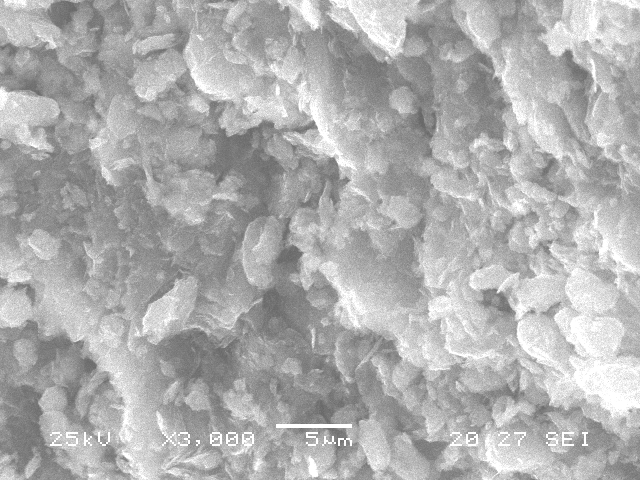  **(c)** | 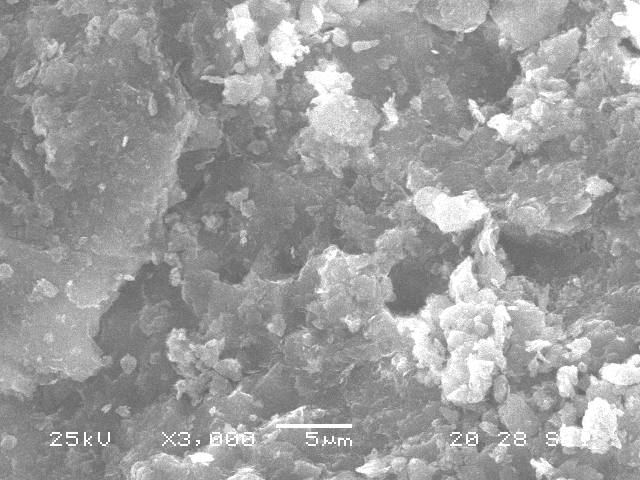 |
| 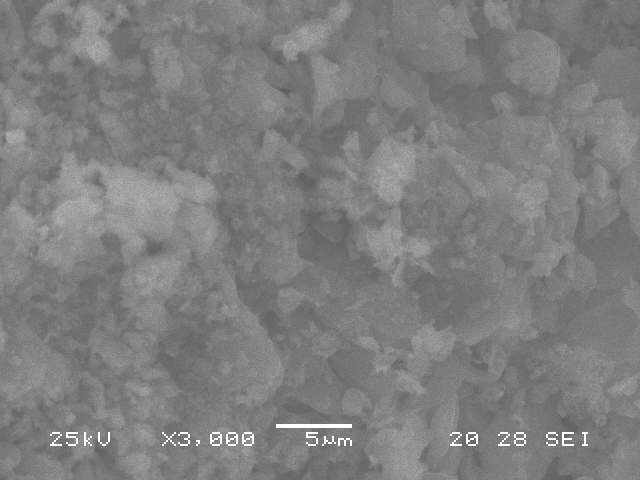  **(e)** | 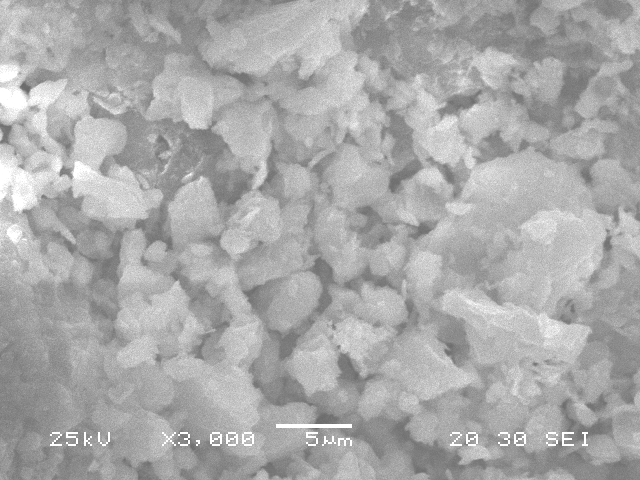  **(f)** |

**Figure 6.** SEM micrographs of the two clay sample SA and CH; (a) and (b) SA and CH crude samples, (c) and (d) heated to 700 oC and (e) and (f) heated to 950 ^o^C.

**1.7.1 Clay features observed using SEM**

Some of the clay features that could be determined from SEM micrographs of the two samples are given in table 13 below.

Table 13. Observed clay features from SEM micrographs of SA and CH clay samples

| Particle size | more than 85 per cent of them less  than 0.5 μm |
| --- | --- |
| Crystallinity | poorly crystallized |
| Clay plates | Very small |
| Aggregates | Spherical artificially formed during spraying and also their size increase with the increase of the final temperature of calcination. |

1. **Experimental Design, Materials, and Methods**

**1.1 Clay fraction isolation**

To separate elements with fraction less than 2 μm oriented preparation were made in 6 different steps; **i**- **Grinding**, it must result in a powder which is not too fine to preserve the clay minerals with a diameter of less than 2 μm [8]. **ii-** **Discharge**, it is done with distilled water, then the sample is subjected to magnetic stirring. The coarse material deposited at the bottom of the flask is removed by decanting. **iii-** **Decarbonation**, this involves the removal of carbonates (CaCO_3_). This operation is necessary for several reasons [9], i) the carbonates include the clay minerals, and hence interfere with their deflocculating; ii) they dilute the clay fraction; obstruct the orientation of the preparations by their non-lamellar form. Hydrochloric acid diluted to 10% is added dropwise to the clay suspension with magnetic stirring in order to avoid local overconcentration, while allowing a little time between each attack. pH is controlled with pH meter. When the solution becomes red, this indicate that the carbonates have been destroyed, at this stage it is necessary to stop the HCl addition and the agitation, then the suspension was allowed to settle. **iv-** **Washing,** its purpose is to free the sample from the excess of the hydrochloric acid, and to allow deflocculating of the clay fraction. If the supernatant becomes clear, it is enough to wash the sample without using the centrifuge, meaning, pour the supernatant, add distilled water, shake, leave it to decant, and so on until a neutral pH is obtained, pH was controlled by litmus paper. Otherwise, the suspensions were centrifuged at 2500 rpm for 5 min. Subsequently, the supernatant is removed, and the precipitate is re-suspended in distilled water. The precipitate is recovered, tested with pH paper, if it is not yet neutral, the centrifugation cycles must be re-instated until the pH is neutral. **v-** **Suspended Sample**, the recovered precipitate is placed in a Beaker of 250 ml, to which distilled water is added, manually shaken, and left to decant for 1h 40mn. If, after 10 minutes, the supernatant is clear. This indicates that deflocculating is poorly performed. For this purpose, one to two drops of ammonia (NH _4_OH) are added which reduces the pH to around 7.0 (the color of the pH paper becomes blue), this is an indication that deflocculating is promoted. **vi-** **Extraction of particles smaller than 2μm**, the supernatant must be cloudy so that the suspension is perfect. The contents of the upper 2 cm of the supernatant are recovered and placed in a Beaker of 100 mL. Distilled water was added and centrifuged at 3500 rpm for 40 minutes. The precipitate obtained is recovered by means of a spatula, deposited in glass slides, left to dry for 24 hours at room temperature and finally passed to analysis by X-ray diffraction.

**1.2 Methylene blue (MB) stain test**

Methylene blue stain test according to AFNOR was carried out using the following procedure [10]; 60.0 g of the clay sample was suspended in 500 mL of distilled water and stirred vigorously until it was homogenized. 5.0 mL of 10.0g/L of methylene blue solution were added to the homogenized solution using a burette. After each addition, spots (stains) were spotted on a Whatman 1441-055 Quantitative Filter Paper Circles, 20 Micron, Grade 41, 55mm Diameter (Figure 6). The sampling and spotting continued until the wet surface surrounding the deep blue spot is turned into light blue color. This represents the saturation of the clay by methylene blue.

The blue value of the clay following AFNOR procedure was calculated using Eq. 6.

| ** | **Eq. 6** |
| --- | --- |

In Eq. 3, V defines the methylene blue volume flowed in mL, 0.01 is the concentration in g/mL of the methylene blue solution, and M is the mass in grams of the dry sample.

The same results of the experiment were used to calculate the ASTM blue index (MIB, in equivalence /100 g) following Eq. 7 [11].

| *$MIB=\frac{\left(E\times V\times0.1\right)}{W}$* | **Eq. 7** |
| --- | --- |

In Eq. 4, E stands for the MB number of equivalents per mL of water, V represents the volume of the MB solution in mL (unit of titration was = 1.0 mL), and W represents the weight of the dry clay sample in g.


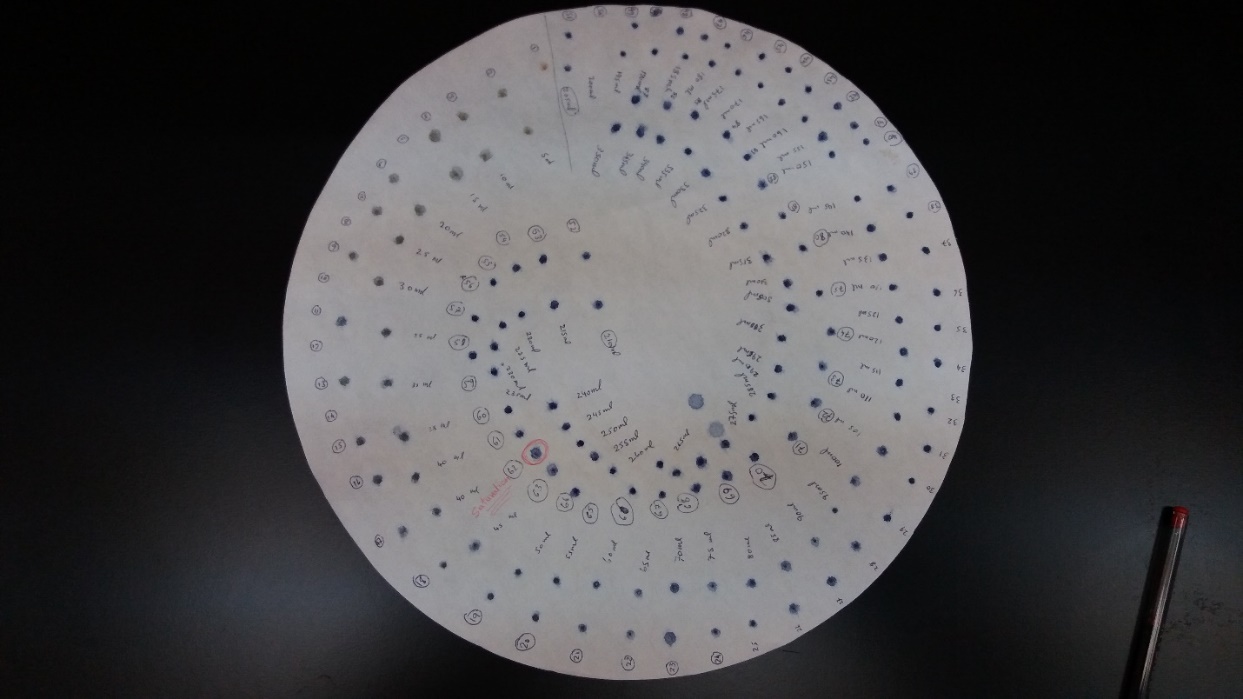


**Figure 6.** Example of Methylene blue stain test

**Acknowledgments**

The authors wish to express their gratitude to the Research Institute of Science and Engineering (RISE), University of Sharjah, Sharjah, UAE for the financial support provided through the seed grant number (1602142018), the Advance Material Science Laboratory, University of Sharjah, for help in running samples and access to equipment.

**References**

[1] Abdelaziz Elgamouz, Najib Tijanib, Ihsan Shehadi, Kamrul Hasan and Mohamad Al-Farooq Kawam. Characterization of a Safi Illite-Kaolinite clay by means of multiple-technique methodology with special focus on methylene blue stain test. Journal of Applied Clay Science, 1.

[2] A. Wassilkowska, A. Czaplicka-Kotas, A. Bielski, and M. Zielina. An analysis of the elemental composition of micro-samples using EDS technique. Czasopismo Techniczne, 2014 (2015) 133.

[3] J.I. Goldstein, D.E. Newbury, J.R. Michael, N.W. Ritchie, J.H.J. Scott, and D.C. Joy, Scanning electron microscopy and X-ray microanalysis, , Springer, 2017.

[4] J. Konopka. Options for Quantitative Analysis of Light Elements by SEM/EDS. (2013).

[5] A. Elgamouz, N. Tijani. From a naturally occurring material (clay mineral) to the production of porous ceramic membranes. Microporous and Mesoporous Materials, (2018).

[6] A. Elgamouz, N. Tijani. Dataset in the production of composite clay-zeolite membranes made from naturally occurring clay minerals. Data in brief, 19 (2018) 2267.

[7] B.J. Saikia, G. Parthasarathy. Fourier transform infrared spectroscopic characterization of kaolinite from Assam and Meghalaya, Northeastern India. Journal of Modern Physics, 1 (2010) 206.

[8] N. El Yakoubi. Potentialités d’utilisation des argiles marocaines dans l’industrie céramique: cas des gisements de Jbel Kharrou et de Benhmed (Meseta marocaine occidentale). (2006).

[9] M.E. Ostrom. Separation of clay minerals from carbonate rocks by using acid. Journal of Sedimentary Research, 31 (1961) 123.

[10] (. Association françaisede Normalization, Mesure de la quantité et de l’activité de la fractionargileuse (Norme Française NF P 94-068), AFNOR, La Défense, Paris, France 1993.

[11] ASTM. Standard test method for methylene blue index of clay. (2009).
